# Lower urinary tract lithiasis of cats in Algeria: Clinical and epidemiologic features

**DOI:** 10.14202/vetworld.2020.563-569

**Published:** 2020-03-26

**Authors:** Hayet Remichi, Fatma Amira Hani, Myriem Rebouh, Chabha Benmohand, Wahiba Zenad, Sofiane Boudjellaba

**Affiliations:** Department of Canine Medicine and General Surgery, Higher National Veterinary School of Algiers, Algeria

**Keywords:** cat, epidemiology, management, radiography, ultrasonography, urolithiasis

## Abstract

**Aim:**

This study aims to describe the clinical symptoms, diagnosis, and treatment of urolithiasis of the lower urinary tract and to determine the main risk factors involved in the occurrence of urinary lithiasis in cats in Algeria from 2016 to 2018.

**Materials and Methods:**

During the study period, 465 cats were examined and 32 cases of urolithiases were selected and investigated by conducting physical examinations, blood analyses, urinalysis, X-ray radiography, and ultrasonography. Parameters such as breed, age, or sex were studied and reported in a farm return to analyze risk factors involved in the formation of lower urinary urolithiasis.

**Results:**

The most clinically relevant symptoms of urolithiasis observed in cats were dysuria, pollakiuria, hematuria, and stranguria. Urinalysis and blood analysis revealed a significant presence of urinary crystals and acute kidney failure in nine cats. The ultrasonography and radiography confirmed the diagnosis of urolithiasis with the incidence of 43.75% and 31.25%, respectively. The lower urinary tract urolithiasis appeared to be more frequent in European and Siamese cats. In addition, cats aged between 4 and 8 years old were the most affected. Male cats (87.50%) were more affected than female cats. Finally, the lower urinary tract urolithiasis was more frequent in cats consuming the commercial pet food, previously castrated, and confined inside the house.

**Conclusion:**

Complete clinical assessments, in addition to complementary examinations, are necessary and beneficial in treating the animal and preventing possible complications. Whether the choice of therapy is surgical or treatment with drugs, it is crucial to understand that the elimination of the stone is not an end, but the beginning of a series of investigations. Because of their impact on both the formation and elimination of metabolites, it has been found that factors, such as race, gender, age, diet, and lifestyle, should be considered as potential risk factors for urolithiasis.

## Introduction

In veterinary medicine, urinary tract diseases are the most common reasons for seeking an examination in cats and dogs. Urolithiasis refers to the development of stones in the kidney, bladder, and/or urethra [1]. These stones, also known as calculi or uroliths, are either mineral or organic in nature. Specific metabolic conditions (e.g., nutritional factors [a protein-rich diet], hydration [lack of water], pH [pH modification], urine volume [low urine volume], hypercalcemia, or hypophosphatemia) influence their formation. Unlike in humans, in domestic carnivores such as cats, uroliths are mainly localized in the lower urinary tract [2]. The previous research has confirmed that the majority of stones in the cat are found specifically in the lower urinary tract [3].

Clinical manifestations are nonspecific; cats can be asymptomatic or have serious disorders negatively affecting their vital prognosis. In practice, the clinician must conduct a full clinical assessment and, if needed, may perform specific complementary examinations to provide better care and to avoid facing possible complications. Factors such as breed, sex, age, diet, urinary tract infections, urinary pH, medical treatments, hydration, litter box, and castration can impact the onset of urolithiasis and composition of the uroliths [4-6]. The first factor is the number of litter boxes available to each cat in the household. The second factor is the size and accessibility of the litter box – finally, the cleanliness of the litter box. For example, a litter box that is difficult to access can motivate the cat to refrain itself, which can cause urolithiasis.

Since the last decade, the frequency of urolithiasis has significantly increased, from 1.5%-8% during the year 1998 to 2003 [7] to 15%-20% during the year 1998 to 2014 [6]. In Algeria, few studies have focused on urolithiasis in the lower urinary tract of cats, and further studies are required to better understand the condition.

Thus, the aim of this study was to describe the clinical signs, diagnosis, and treatment of lower urinary tract urolithiasis in cats in Algeria and identify the main risk factors associated with the disease.

## Materials and Methods

### Ethical approval

The investigations protocol was based on the clinical examination of cats seen at the Department of Canine Medicine and General Surgery of the Higher National Veterinary School of Algiers. Hence, ethical approval was not required.

### Animals

Between 2016 and 2018, 465 cats were examined at the Department of Canine Medicine and General Surgery of the Higher National Veterinary School of Algiers. Thirty-two cases of urolithiases were selected and investigated by conducting a physical examination, blood analysis, urinalysis, radiography, and ultrasonography.

### Physical examination

Clinical signs help localize the problem to the lower urinary tract. Information from the animal owners helps to determine the duration and severity of symptoms. Physical examination includes evaluation of body temperature, heart rate, respiratory rate, mental state, mucous membranes color, capillary refill time, cardiopulmonary auscultation, and abdominal palpation.

### Urinalysis and blood analysis

Chemical and biochemical analyses on urine samples revealed the urinary pH, color, turbidity, urine specific gravity, presence of blood, protein, hemoglobin, and presence of crystals. Urine samples were obtained by cat catheterization, or during urination. Urinalysis consisted of physicochemical analysis by reagent strips (Centrivet), urine density by refractometer, and urinary sediment after centrifugation for 5 min by optical microscopy.

The analyzed blood parameters were the blood urea nitrogen (BUN), the blood creatinine, and the blood kalemia. Blood samples were taken aseptically from the cephalic or jugular veins in dry tubes and centrifuged.

### Imaging investigations

Radiographic and ultrasonographic imaging methods were applied to cats without sedation or anesthesia. The size, number, shape, and location of calculi were determined and surgical procedures were carried out under general anesthesia.

Abdominal radiography was performed using animal X-ray machine (VET-TECH by GeR) Radiographs were processed using a computed radiography system. The radiographs were taken on a ventral dorsal or lateral view.

In this work, ultrasonography of all cats was performed using a real-time scanner (Titan Sonosite) with a 7.5-10 MHz broadband, curvilinear probe. The animals were positioned on lateral or dorsal recumbency, scanning area was shaved, and ultrasonic gel applied to the skin. During the ultrasound, all parenchymas of the kidney were evaluated; the size, number, shape, and location of calculi were determined.

### Calculi management

Therapeutic decisions depend on the physical condition of the animal, the type, size, and location of the calculi. Surgical procedures were carried out under general anesthesia. Uroliths were removed by urethrostomy or cystotomy.

### Observation of the risk factors

A standardized survey was established, bearing different parameters and their influence on the formation of urolithiasis: Breed, age, sex, castration, diet, hydration, cleanliness, motives of consultation, physical signs, and diagnostic means. The study data were analyzed in a Microsoft Office 2013 Excel spreadsheet, in which the percentages of each criterion studied were calculated.

A farm return was established, bearing different parameters and their influence on the formation of urolithiasis: Farm return represents breed, age, sex, castration, diet, hydration, cleanliness, motives of consultation, physical signs, and diagnostic means.

## Results

A total of 32 cats presented to the Department of Canine Medicine and General Surgery suffering from urolithiasis based on their medical history of abdominal distension (nine cases; 28.00%) and urinary disorders and vomiting (30 and 12 cats; 93.75%, and 37.5%, respectively). From these 32 cats, a loss of appetite was reported in 25 cases (78.12%) and a loss of weight was reported in ten cases.

The major clinical symptoms of affected cats with urolithiasis are presented in Table-1. Dysuria, pollakiuria, strangury (30 cases; 93.57%), hematuria (28 cases; 87.5%), dehydration (15 cases; 46.88%), distended bladder (14 cases; 43.75%), and restless (six cases; 18.75%) were observed.

**Table-1:** Clinical signs, urinalysis, and biochemical blood analysis.

Clinical signs and physical findings	n (%)
Dysuria, pollakiuria, stranguria	30 (93.57)
Hematuria	28 (87.56)
Dehydration	15 (46.88)
Distended bladder	14 (43.75)
Inappropriate urination, urge incontinence	6 (18.75)

**Urinalysis and biochemical blood analysis**	**n (%)**

pH acidic	21 (65.62)
pH alkaline	11 (34.37)
Blood	28 (87.5)
Leukocytes’	12 (37.5)
Protein	6 (18.75)
Epithelial cells	18 (56.25)
Crystals	32 (100)
Specific gravity <1.038	13 (40.62)
Blood urea nitrogen	9 (28.12)
Blood creatinine	9 (28.12)
Hyperkalemia	6 (18.75)

The urinalysis revealed: Acidic urine pH (21 cases; 65.62%), alkaline urine pH (11 cases; 34.37%), urine blood presence (28 cases; 87.5%), urine protein presence (six cases; 18.75%) (1.0 g/l and 3.0 g/l), leukocytes presence (12 cases; 37.5%), specific gravity (< 1.038) (13 cases; 40.62%), presence of epithelial cells (18 cases; 56.25%), and presence of crystals in all affected cats (100%) (Table-1). Nine cats with urolithiasis (28.12%) had an elevation of BUN and creatinine. Six cats with urolithiasis (18.75%) had hyperkalemia (Table-1).

**Figure-1:**
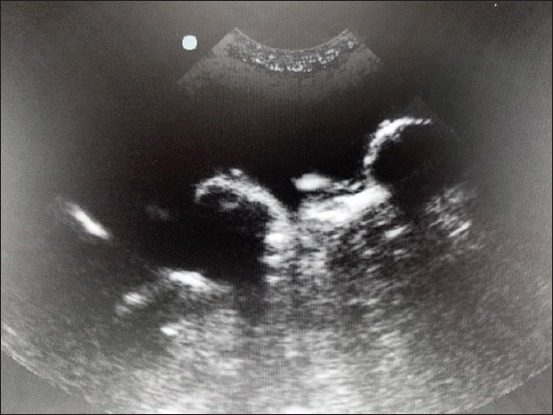
Ultrasonographic view of vesical uroliths showing shadow cones.

**Figure-2:**
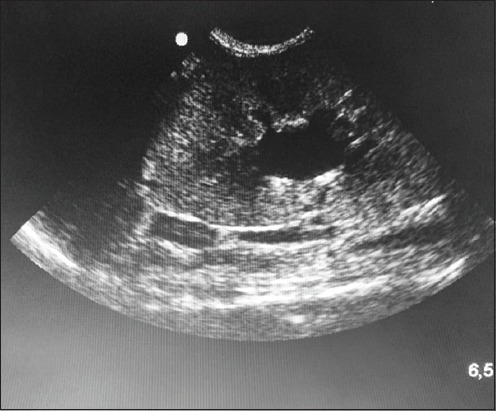
Ultrasonographic view of hydronephrosis in a cat.

**Figure-3:**
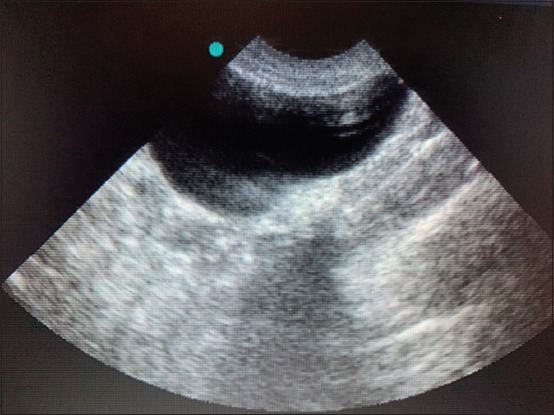
Ultrasonographic image of the bladder showing urinary sand.

**Figure-4:**
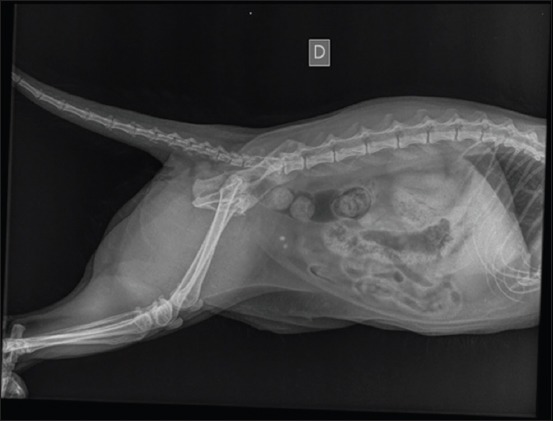
Standard radiographic image (lateral decumbency) in a cat showing two bladder stones.

The 32 cases were eligible for both ultrasonogram and X-ray radiography. The ultrasonography was used to diagnose 14 cases of urolithiasis (43.75%); 12 uroliths were localized in the bladder, and two were in the urethra. Ultrasonographic images of uroliths were identified by a limited hyperechogenicity, accompanied by a shadow cone (Figure-1). Hydronephrosis was diagnosed in three cases (9.37%); the ultrasound imaging determined a widening of the renal pelvis, the renal diverticulum, and the ureter (Figure-2). Ultrasonography allowed the visualization of the distended urinary bladder with cystitis (12 cats; 37.5%) and the urinary bladder sand (one case; 3.13%) (Figure-3). Radiography was used to confirm the presence of stones (ten cats; 31.25%). Overall, the studied cases of lithiasis were radiographically opaque (Figure-4).

**Figure-5:**
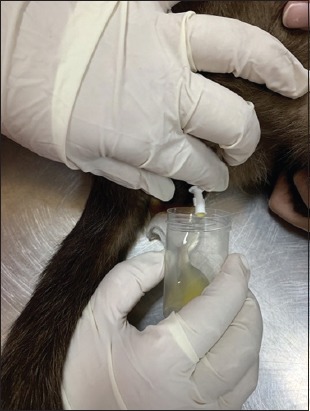
Catheterization in feline urethral obstruction.

**Figure-6:**
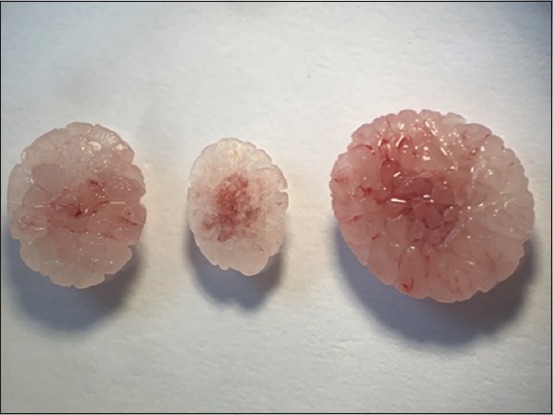
Struvite uroliths removed after cystotomy.

**Figure-7:**
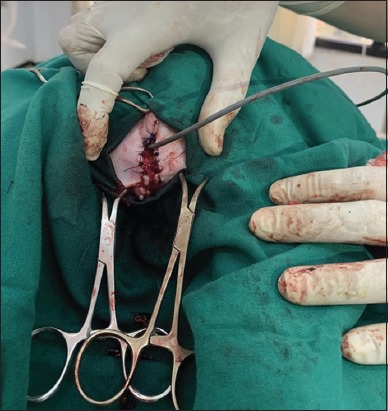
Obstruction in a cat perineal urethrostomy.

**Figure-8:**
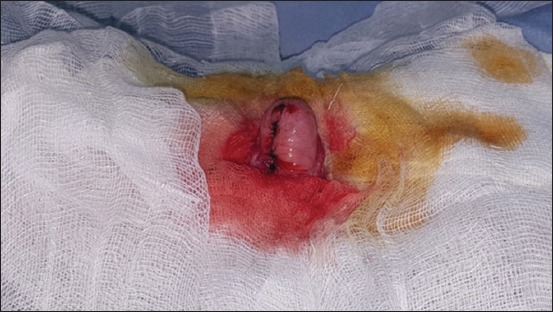
Cystotomy in a cat presenting bladder stones.

Urinary catheterization (Figure-5) was conducted in 14 cats (43.75%) suffering from an obstructive syndrome. Uroliths were removed (Figure-6), by urethrostomy in four cases (Figure-7) and by cystotomy in two cases (Figure-8). Medical treatment was prescribed in 12 cats (37.5%) with cystitis.

Among the ill cats, 21 cats were of European breed (65.62%), eight cats were of the Siamese breed (25%), two cats were of Turkish Angora breed (6.25%), and one cat was of cross-breed (3.25%) (Table-2).

**Table-2:** Epidemiological parameter of study.

Settings	n (%)
Breed	
European shorthair	21 (65.62)
Siamese	8 (25.00)
Angora	2 (6.25)
Cross-breed	1 (3.13)
Age (year)	
<2	3 (9.38)
3-4	11 (34.38)
5-8	17 (53.13)
9-12	1 (3.13)
Gender	
Male	28 (87.50)
Female	4 (12.50)
Castration	
Un-castrated	9 (28.12)
Castrated	23 (71.81)
Living conditions	
Indoor	26 (81.25)
Outdoor	6 (18.75)
Diet	
Dry commercial pet food	21 (65.63)
Homemade food	11 (34.38)

A total of 17 lithiasis cats (53.13%) belonged to the age group of 4-8 years old, 11 cats to the age group of 2-4 years (34.38%), three cats were under 2 years old (9.38%), and only one cat was 8 years old (3.13%) (Table-2).

Thus, this study showed a high percentage of male cats with urolithiasis (28; 87.50%); among them, 17 cats (53.12%) have developed an obstructive syndrome. Four females (12.50%) were concerned by urolithiasis, on the other hand (Table-2). In addition, 23 castrated cats (71.87%) and nine un-castrated cats (28.12%) were affected (Table-2). Indoor lifestyle was reported in 26 cats with urolithiasis (81.25%), whereas only six (18.75%) cats having access to outdoors were afflicted by the disease (Table-2). In the studied population, 21 animals (65.83%) consumed commercial pet food, and 11 cats (34.38%) had a homemade diet (Table-2).

## Discussion

Clinical examination effectively allows for the diagnosis of clinical symptoms and the treatment of the lower urinary urolithiasis of domestic cats. In this study, signs of urolithiasis are associated with inflammation or infection of the urinary tract. Our clinical tests revealed hematuria, pollakiuria, and strangury as the most common symptoms of urolithiasis. In cases, where urine flow is completely obstructed, other symptoms such as vomiting, anorexia, and dehydration were observed, due to acute kidney failure. These findings are in coordination with the previous studies in cats suffering from lower urinary tract urolithiasis [8-10].

Our findings support the notion that imaging, urinalysis, and blood analysis allow for effective diagnosis of urolithiasis [11,12]. Furthermore, radiography is generally the first complementary exam used to confirm the presence of stones [12,13]. In this study, lithiasis is detected radiographically in ten cases. However, visualizing lithiasis on radiography without preparation will depend on their sizes, their localizations, and their radio-opacity. These parameters help to determine the nature of the stones [14]. Only radio-opaque uroliths, such as calcium phosphate stones, oxalate, and ammonia magnesium phosphate uroliths over 2 mm, can be radiographically detected [15] whereas, urate stones are not radiographically identified and required double-contrast radiography to visualize them [11,13].

Lithiasis was identified ultrasonographically by their hyperechogenic aspect, with the presence of rear acoustical shadow [16]. Different authors agree on the fact that the sonographic appearance depends on the density and the composition of the stone. When the selected sensor frequency is too low or, if the uroliths are not directly on the path of the ultrasound beam, the shadow cones associated with the calculi are either unclear or absent [17].

Hydronephrosis was observed in five cats and characterized by an anechoic aspect. Furthermore, dilatation of the renal pelvis with an increase of the kidney size contributed to pathology [16,18]. Cystitis was diagnosed in 12 cats. It is defined by a hyperechoic parietal thickening of the membrane. Cystitis can cause stone formation. The presence of the germs responsible for cystitis can influence the urinary pH and promotes the formation of stones. However, it is interesting to note that cystitis can also be a consequence of urolithiasis due to contact of the calculus with the bladder membrane. Consequently, additional research can bring clarification regarding the origin of cystitis. The sensitivity of ultrasonography for detecting ureteral calculi is 77% and can be increased to 90% while combining ultrasonography and radiography [13,15].

A few urinary disorders were detected when diagnosing urolithiasis such as changing of pH, blood urine presence, and presence of germs in the urine. The urine specific gravity represents the ratio of the urine density to the water density, which allows determining how well the kidney is functioning. The existence of large quantities of crystals in urine is an indicator of the over-saturation of the urine but not of the existence of urolithiasis. Urine specific gravity test and urine pH can help to suggest which type of calculus is present and/or detect the presence of urinary tract infections. Calcium oxalate and struvite uroliths are the most commonly identified uroliths in cats [19,20]. Urinary pH is dependent on the type of food in cats. In the case of calcium oxalate uroliths, it is interesting to mention that the food should have an average tenure in calcium, oxalate, and phosphorus to prevent the occurrence of the uroliths consequently.

Struvite uroliths occur when urinary pH is too basic. A diet favoring an average acidic pH and limiting the minerals contributing to the formation of struvite can be recommended.

Kidney failure was diagnosed on the basis of an elevation of BUN and serum creatinine concentration. In the population of cats evaluated, only nine had a kidney failure. The results did not conclude to a positive association between urolithiasis and kidney failure [20].

The therapeutic decisions in our study were multiple. In fact, they decided accordingly to the stage of the disease. To re-establish normal urine flow, surgery was sometimes an unavoidable solution. A cystotomy is the most common uroliths surgery .Some uroliths may also be dissolved with diet, or with medical, and prophylactic treatments [21]. Therapeutic decisions depend on the physical condition of the animal, the type, size, and location of the calculi. Our findings have been coherent with those set out by Brissot and Bouvy [22], Osborne *et al*. [23], Kamiloğlu and Kiliçoğlu [24].

This study revealed that long-haired cats (European breeds) were more affected than short-haired (Siamese). For instance, this opens an interesting discussion about genetics and breeders being aware of purebred cats having potential difficulties. It is recommended that the cat’s owners of these breeds favor a diet and hygiene, limiting the formation of urolithiasis. It is essential to communicate and also raise awareness of the risk factors linked to this genetic aspect.

All feline breeds can be affected by lithiasis [1]. Appel’s *et al*. [25] study identified significant associations between breed and uroliths composition. The affection was more observed in males compared to females. Some researchers showed that males cats would be more susceptible to present lithiasis than females [23,26]. The narrow and long urethra of male cats makes them more likely than female cats to develop obstruction [19,27]. The literature did not report that the age factor influences the occurrence of lithiasis, but certain types of stones were detected in adult cats, and others were identified in the youngest ones [28,29]. Syme [2] had concluded that age, sex, and breed were risk factors for urinary stone formation. These results revealed similarity with the bibliographic data [23,29,30]. Cats who have access to outdoors are less affected than those living indoors [19]. Urolithiasis occurrence in cats depends on the animal’s activity, the use or no of a litter box, and the lifestyle (outdoor or indoor) [19]. These conditions motivate the animal to refrain itself, which favors urine stasis and saturation, and increases the risk of formation of stones, or urinary tract infections [20]. Animals affected were those given commercial pet food. Several authors report that the proportion of urolithiasis is higher in cats consuming only dry food [27,30]. Food is a leading factor in the onset of urinary stones in the cat: Diet content influences urinary pH, which may increase the risk of lithiasis formation [1,4,26]. According to Bartges *et al*. [31], food was essential in the prevention of urinary lithiasis. It is also reported that some diets can reduce urinary concentrations and thus, favor growth inhibitors activity and diminish crystals aggregation in the case of calcium oxalate lithiasis. A cat is an animal that drinks little amounts that can further aggravate the case [32]. Palm and Westropp [33] recommend to give the cats wet food and to increase water taking enriched with sodium. Treatment and prophylaxis necessitate stamping out factors favoring the formation of lithiasis when they are known.

## Conclusion

Urolithiasis should not be seen as a single problem but as a consequence of various disorders. In practice, the clinician should carry out a complete clinical assessment and requires specific complementary examinations if necessary, to better treat and cope with possible complications. Therapeutic decisions are made in relation to the physical condition of the animal and the consequences of urolithiasis. Factors, such as breed, gender, age, diet, and lifestyle, are considered risks because they are involved in the formation and elimination of uroliths. These decisions may be hygienic, medical, or surgical. Thus, it is necessary to understand that the elimination of the stones does not signify the end of treatment; rather, the beginning of a series of investigations to identify more effective prevention and therapy protocols.

## Authors’ Contributions

HR: Conceived and designed the study; achieved the imaging procedures; medical consultation, and writing of the article. FAH: Responsible for the sample and data collection. MR, CB, and WZ: Undertook the surgical procedures and carried out the laboratory exams. SB: Data analysis and guidance in the writing of the manuscript. All authors read and approved the final manuscript.
